# Don't break a leg: running birds from quail to ostrich prioritise leg safety and economy on uneven terrain

**DOI:** 10.1242/jeb.102640

**Published:** 2014-11-01

**Authors:** Aleksandra V. Birn-Jeffery, Christian M. Hubicki, Yvonne Blum, Daniel Renjewski, Jonathan W. Hurst, Monica A. Daley

**Affiliations:** 1 Structure and Motion Laboratory, Royal Veterinary College, Hawkshead Lane, Hatfield, Hertfordshire AL9 7TA, UK; 2 Dynamic Robotics Laboratory, Oregon State University, 204 Rogers Hall, Corvallis, OR 97331, USA

**Keywords:** Bipedal running, Gait stability, Ground birds, Injury avoidance, Trajectory optimisation

## Abstract

Cursorial ground birds are paragons of bipedal running that span a 500-fold mass range from quail to ostrich. Here we investigate the task-level control priorities of cursorial birds by analysing how they negotiate single-step obstacles that create a conflict between body stability (attenuating deviations in body motion) and consistent leg force–length dynamics (for economy and leg safety). We also test the hypothesis that control priorities shift between body stability and leg safety with increasing body size, reflecting use of active control to overcome size-related challenges. Weight-support demands lead to a shift towards straighter legs and stiffer steady gait with increasing body size, but it remains unknown whether non-steady locomotor priorities diverge with size. We found that all measured species used a consistent obstacle negotiation strategy, involving unsteady body dynamics to minimise fluctuations in leg posture and loading across multiple steps, not directly prioritising body stability. Peak leg forces remained remarkably consistent across obstacle terrain, within 0.35 body weights of level running for obstacle heights from 0.1 to 0.5 times leg length. All species used similar stance leg actuation patterns, involving asymmetric force–length trajectories and posture-dependent actuation to add or remove energy depending on landing conditions. We present a simple stance leg model that explains key features of avian bipedal locomotion, and suggests economy as a key priority on both level and uneven terrain. We suggest that running ground birds target the closely coupled priorities of economy and leg safety as the direct imperatives of control, with adequate stability achieved through appropriately tuned intrinsic dynamics.

## INTRODUCTION

Modern bird success is commonly attributed to flight, but could equally be ascribed to a heritage of bipedal agility tracing back 230 million years to theropod dinosaurs. Ground birds, such as quail and ostriches, move with speed and economy through complex natural terrain environments (Alexander et al., 1979; Birn-Jeffery and Daley, 2012; Dial, 2003; Jindrich et al., 2007; Rubenson et al., 2004). These athletes span the broadest body mass range among extant bipeds, over 500-fold from quail to ostrich. Birds thus provide a natural animal model for understanding the functional demands of striding bipedalism and how these demands change with body size (Gatesy and Biewener, 1991; Hutchinson and Garcia, 2002; Roberts et al., 1998a).

Here, we ask two questions fundamental to locomotor behaviour: (1) what are the task-level leg control priorities of running animals; and (2) how do these priorities vary with terrain and body size? Running animals must control their legs to balance numerous, sometimes conflicting, task-level demands including minimising energy cost (Cavagna et al., 1977; Roberts et al., 1998a; Srinivasan and Ruina, 2006), avoiding falls (Clark and Higham, 2011; Daley and Usherwood, 2010), maintaining desired speed and direction (Birn-Jeffery and Daley, 2012; Carrier et al., 2001; Daley and Biewener, 2006; Jindrich and Full, 2002), and avoiding injury from excessive leg forces (Biewener, 1989). Many features of steady locomotion emerge from minimising muscle work and energy cost, suggesting economy as a key priority (Cavagna et al., 1977; Kram and Taylor, 1990; Srinivasan and Ruina, 2006). Yet, steady locomotion is likely rare in the complex topographies of natural environments (Nishikawa et al., 2007). Therefore, priorities might sometimes shift away from economy, particularly in non-steady behaviours, because animals must avoid catastrophic falls and injury to survive.

The ideas above suggest that locomotor stability might be among the key control priorities of animals, yet stability remains poorly understood and challenging to measure. ‘Avoiding falls’ has potential as an ultimately relevant and general definition of stability, because falls increase risk of predation. Yet, falls can be preceded by musculoskeletal injury from repetitive high-stress loading (Verheyen et al., 2006). Thus, both instability and injury can be the proximate causes of falls, so body stability and leg injury avoidance have the potential to be key and distinct priorities.

Here we use ‘body stability’ to refer to attenuating deviations in body centre of mass (CoM) motion from steady gait, as opposed to the looser definition of general stability as ‘avoiding falls’. Mathematical analyses of body stability (local or cyclical asymptotic stability) focus on whether gait perturbations diminish over time, returning the body CoM to the nominal periodic gait (Blickhan et al., 2007; Blum et al., 2011; Dingwell and Kang, 2007; Geyer et al., 2005; Seyfarth et al., 2003). For example, Poincaré sections have been used to observe the deviations of the body CoM states at apex height (Blum et al., 2011; Geyer et al., 2005). Empirical evidence suggests that running animals do exhibit stable body motion, recovering from unexpected perturbations within approximately two to three steps (Daley and Biewener, 2011; Jindrich and Full, 2002).

Pinpointing the underlying mechanisms used by animals to achieve stability is also challenging, because significant interplay occurs between intrinsic musculoskeletal dynamics and active neural control. Here, we conceptually distinguish between stability as a control priority, a direct objective of the applied active control, and intrinsic-dynamic stability, properties conferred by the inherent dynamics without neural feedback control. Intrinsic stability mechanisms can be revealed by subjecting an animal to a sudden, unexpected perturbation, to observe the immediate response in the short time period before feedback is possible (Daley et al., 2009; Full et al., 2002; Jindrich and Full, 2002). Animals employ intrinsic stability mechanisms to minimise gait disturbances and facilitate rapid recovery from surprise perturbations (Daley and Biewener, 2006; Jindrich and Full, 2002). This minimises the need for active control intervention. Yet, it remains unknown whether running animals rely heavily on intrinsic stability mechanisms even for anticipated terrain changes, or also target body stability through active control. Active mechanisms may include anticipatory manoeuvres to minimise the initial effects of a terrain change on body trajectory, and reactive responses to return the body toward steady gait once perturbed.

There are several lines of evidence to suggest that animals might actively target body stability as a control priority, even on uneven terrain: (1) previous perturbation experiments have demonstrated that animals minimise deviations from steady body CoM dynamics and recover quickly to steady gait (Daley and Biewener, 2006; Farley et al., 1998; Ferris et al., 1999; Grimmer et al., 2008; Jindrich and Full, 2002; Moritz and Farley, 2003), (2) hopping and running humans target steady body CoM trajectory on variable terrain, and use active mechanisms to do so under ‘expected’ conditions (Ferris et al., 1999; Ferris et al., 1998; Grimmer et al., 2008; Moritz and Farley, 2003; Moritz and Farley, 2006; Moritz et al., 2004), and (3) animals have been observed to allow variance in joint dynamics while minimising variance in body CoM trajectory (Chang et al., 2009; Yen et al., 2009). These findings have led to the suggestion that steady body CoM trajectory is a direct target of neural control (Chang et al., 2009; Ferris et al., 1999; Moritz and Farley, 2003; Moritz and Farley, 2006; Yen et al., 2009). Additionally, minimising fluctuations in body CoM trajectory has the potential to minimise external mechanical work, which factors into the energy cost of locomotion (Srinivasan and Ruina, 2006). Nonetheless, uneven terrain locomotion has been studied in relatively few animals and terrain conditions; thus, it remains unclear whether animals prioritise steady CoM trajectory over a wide range of species and terrain contexts.

**Fig. 1.**
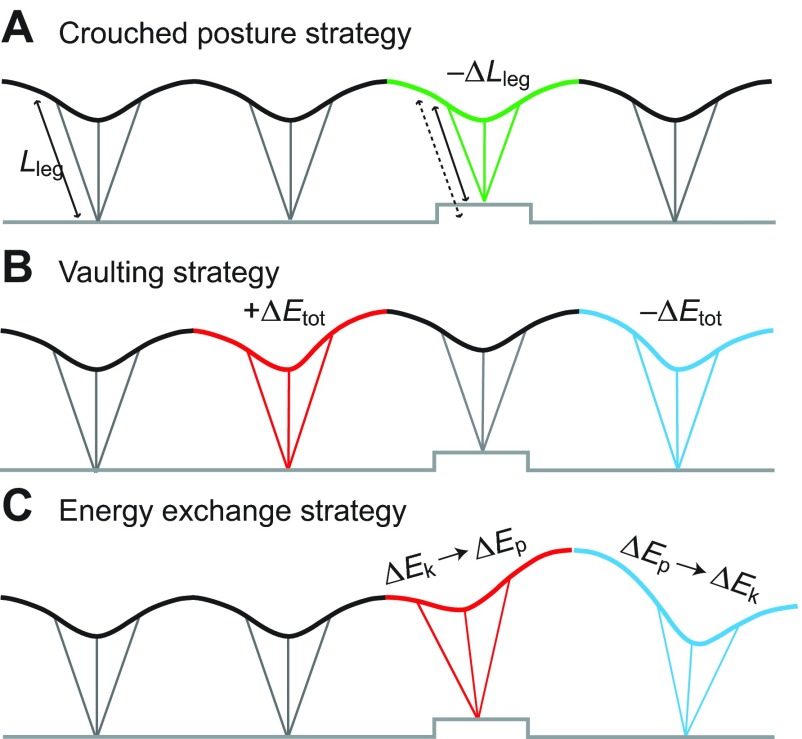
**Obstacle negotiation strategies as a ‘window’ into task-level control priorities.** Schematic illustration of idealised strategies, as hypothetical reference points. (A) Adopting a crouched leg posture on the obstacle could allow constant body motion, minimising deviations in body centre of mass (CoM) trajectory and mechanical energy, but would increase muscle force required to support body weight, due to altered gearing. (B) Vaulting onto the obstacle can maintain typical leg posture and body trajectory on the obstacle step, but requires work to increase mechanical energy (*E*_tot_) in step −1. (C) An additional possible strategy that can occur without anticipatory adjustments is exchange between kinetic energy (*E*_k_) and gravitational potential energy (*E*_p_). This can occur because of altered landing conditions in the obstacle step [*E*_k_→*E*_p_ (Daley and Biewener, 2011)], in the obstacle dismount [*E*_p_→*E*_k_ (Birn-Jeffery and Daley, 2012)] or both. It is also possible to use a combination of multiple strategies.

Here we study running dynamics of birds negotiating a visible, single-step obstacle (Fig. 1), which allows the birds to plan their strategy, from which we can infer task-level control priorities. The single-step obstacle puts demands for body stability (attenuating deviations in body trajectory) into direct conflict with demands to regulate leg posture and leg loading, which influence musculoskeletal loads and thus both economy and leg safety. Active manoeuvres to negotiate an obstacle fall between two hypothetical extremes: ‘crouching’, which minimises fluctuations in body trajectory (Fig. 1A), and ‘vaulting’, which maintains consistent leg force–length dynamics (Fig. 1B). In a ‘crouching’ strategy, birds shorten the leg to accommodate the obstacle, using posture change to minimise deviations in body trajectory from steady gait, which also minimises external mechanical work. However, crouched posture demands increased muscle forces because of changes in leg posture and musculoskeletal gearing (Biewener, 1989; Daley and Biewener, 2011; McMahon et al., 1987). Changes in leg posture and loading have significant implications for the metabolic energy cost of locomotion, because cost depends on muscle force (Kram and Taylor, 1990;

McMahon et al., 1987) and work (Srinivasan and Ruina, 2006). In a ‘vaulting’ strategy, the bird launches upwards in anticipation of the obstacle (Fig. 1B), actively deviating from steady gait to raise body height onto the obstacle and minimise fluctuations in leg posture and loading (Birn-Jeffery and Daley, 2012). We suggest that a greater degree of ‘crouching’ reflects a priority to stabilise body CoM trajectory and minimise external work, whereas ‘vaulting’ reflects a priority to maintain consistent musculoskeletal force–length dynamics, for effective gearing and leg safety. Thus, this terrain perturbation allows us to test the hypothesis that stability of body CoM trajectory is a direct priority of active locomotor control.

To also test the hypothesis that control priorities shift between body stability and leg safety with increasing body size, we compare obstacle negotiation behaviour among birds from bobwhite quail (0.22 kg) to ostrich (117 kg), spanning a 500-fold mass range. This hypothesis is based on the idea that non-steady locomotor behaviours may reflect use of active control to overcome the inherent challenges of body size: large animals are at high risk of injury because of high musculoskeletal stresses, whereas small animals are less injury prone (Vogel, 1981), but live in ‘rougher’ terrain relative to their leg length. Large animals are limited by the strength of their legs because peak loads increase with body mass, but strengths of musculoskeletal structures increase with cross-sectional area (Biewener, 1989). To ameliorate this problem, large animals run with straighter legs to minimise muscle and bone stresses (Biewener, 1989; Gatesy and Biewener, 1991). Nonetheless, large animals face an inherently high risk of injury because changes in leg posture or loading [ground reaction forces (GRFs), leg touch-down

**Fig. 2.**
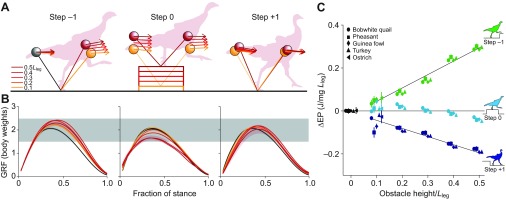
**Observed obstacle negotiation strategy.** Ground birds from bobwhite quail to ostrich use similar dynamics to negotiate obstacles, reflecting a consistent combination of vaulting onto the obstacle (step −1), some crouching on the obstacle (step 0) and energy exchange in the dismount, converting *E*_p_ to *E*_k_ (step +1). (A) Dynamics illustrated by velocity vectors (arrows), CoM position (balls) and leg posture (stick figures). (B) Ground reaction force (GRF). (C) Change in potential energy per step during obstacle negotiation. A and B show a grand mean across species for the aerial running data (walking and grounded running trials excluded from the average trajectories). In step 0, crouching accounts for 39±7% (mean ± s.d.) of the obstacle height across species. Peak forces remain within 0.35 body weights (BW) of level gait (B; grey shading indicates ±0.5BW). See supplementary material Tables S1–S6 for detailed statistics.

collisions] could increase muscle and bone stresses beyond safety factor limits. In contrast, small animals run with crouched (flexed) leg posture (Biewener, 1989; Gatesy and Biewener, 1991), which allows smooth body motion with robustness to terrain variation (Daley and Usherwood, 2010; McMahon and Cheng, 1990). Thus, we aim to use the spectrum of ground bird body size and leg posture as a window into the relationship between control priorities, morphology and terrain conditions. We reason that control priorities and morphology may have co-evolved to overcome the consequences of scaling. We therefore predicted that small animals would prioritise body stability, using postural changes to minimise gait deviations to a greater degree than larger animals. We expected large animals to prioritise leg injury avoidance, minimising changes in leg posture and peak forces.

## RESULTS

**Fig. 3.**
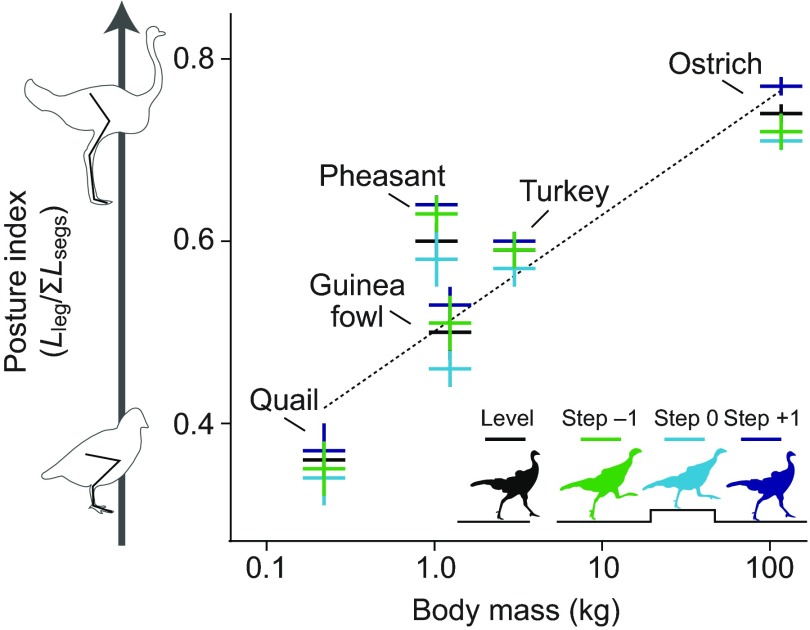
**Scaling of leg posture in level and 0.1 times nominal leg length (*L*_leg_) obstacle terrain.** Leg posture is measured as a ‘posture index’ equal to the ratio of hip height to the sum of the hindlimb segment lengths (Gatesy and Biewener, 1991), measured at mid-stance during running. Despite differences among species in leg posture during steady gait, all species used a similar range of posture in uneven terrain. Contrary to predictions, small birds did not make greater use of postural changes to stabilise body trajectory.

Surprisingly, we discovered that all species, regardless of body size, used a dynamically similar behaviour. The birds negotiated the obstacle over three steps (Fig. 2A), performing an anticipatory vault onto the obstacle in step −1, maintaining a nearly symmetric stance on the obstacle (step 0), but with a significantly more crouched posture (supplementary material Tables S2, S5), and dismounting the obstacle using an ‘energy-exchange’ strategy (step +1). Birds used a consistent balance of ‘vaulting’ and ‘crouching’, with crouching on the obstacle accounting for 44% of obstacle height for the 0.1*L*_leg_ obstacle, where *L*_leg_ is nominal leg length (supplementary material Table S2, *H*_TD_, level versus step 0=−0.044), which did not differ significantly across species (Fig. 3; supplementary material Table S3, ‘step 0’). In the obstacle dismount (step +1; Fig. 2A), birds landed with a steeper leg angle, avoiding high peak forces and converting gravitational potential energy, *E*_p_, to forward kinetic energy, Δ*E*_k_, during stance (Δ*E*_p_, Δ*E*_k_; supplementary material Tables S2, S5). The step +1 dynamics, and in particular the steeper leg contact angle, similar peak force and exchange of *E*_p_ to forward *E*_k_, is qualitatively similar to the dynamics of birds negotiating an unexpected pothole or a visible downward step (Blum et al., 2014; Daley and Biewener, 2006).

Deviations from steady gait during obstacle negotiation scaled similarly across species, once normalised to dimensionless quantities based on body mass, gravity and isometric leg length scaling (see Materials and methods for calculation). Small birds did run with more ‘crouched’ postures on average during their nominal steady gait (Fig. 3), consistent with previous findings (Gatesy and Biewener, 1991). Yet, small birds did not make greater use of postural change during obstacle negotiation to minimise change in body dynamics (Fig. 3). We observed no significant trends with

**Fig. 4.**
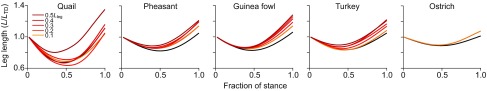
**Virtual leg length trajectories for each species measured in the pre-obstacle step (step −1).** In obstacle terrain, leg extension in the second half of stance increases with obstacle height, across species. Although quail exhibit greater variation in leg extension, they exhibit a similar trend of increasing leg extension with obstacle height. Quail did not differ significantly from other species in the magnitude of *E*_p_ increase during step −1 (supplementary material Tables S3, S6), so the difference in leg trajectory reflects greater co-variance in leg angle and length, not a difference in overall body dynamics.

body size on obstacle negotiation strategy (Fig. 2C; supplementary material Tables S1–S6). The evidence therefore refutes the hypothesis that active control priorities shift significantly with body size. Thus, although the crouched posture of small animals may provide intrinsic stability against unexpected disturbances (Daley and Usherwood, 2010), this does not imply body stability as a task-level control priority.

Across species, the degree of crouching on the obstacle remained consistent with increasing obstacle height, accounting for 39±7% (mean ± s.d.) of obstacle height across conditions (supplementary material Table S5, step 0, *H*_TD_). Deviations in leg posture and body dynamics did increase in magnitude with obstacle height (supplementary material Table S5), but reflected a consistent balance between ‘vaulting’ and ‘crouching’ strategies, with no evidence of a breakpoint or shift in strategy with increasing obstacle height (Fig. 2C). The observed behaviour is inconsistent with body stability as a dominant control priority, and instead appears to reflect the influence of competing demands. Consequently, we focus our analysis below on interpreting the data with respect to alternative possible priorities, such as injury avoidance and energy economy.

Force trajectories remain remarkably similar to level running (Fig. 2B), with peak GRF within 0.35 body weights (BW) of the level mean, even during negotiation of obstacles up to 0.5*L*_leg_ (supplementary material Table S5). The largest shifts in peak force occurred in step −1, during the anticipatory manoeuvre (Fig. 2B), and were statistically significant for obstacle heights 0.2–0.5*L*_leg_ (supplementary material Table S5). The loading phase of the force profiles retained a profile similar to that observed on level terrain. Force profiles deviated from the level terrain trajectory in the unloading phase, or second half of stance. A pronounced asymmetry in the GRF was apparent across species and conditions (including level terrain), with peak GRF at 20–45% of stance (Fig. 2B).

In addition to consistent leg forces, we observed similar leg actuation trends across terrains and species. Birds added energy through leg extension during leg unloading, with an asymmetric profile corresponding to the asymmetry in force (Fig. 4). In obstacle terrain, the vaulting behaviour in step −1 was achieved by increasing force and leg extension during the latter half of stance (Fig. 2B, Fig. 4). Quail exhibited more variance than other species in the leg length trajectory (Fig. 4). Yet, the net change in *E*_p_ during the pre-obstacle step did not significantly differ between quail and other species (supplementary material Tables S3, S6, step −1, Δ*E_P_*). Instead, the variance in leg length trajectory in quail appears to reflect higher co-variance between leg length and leg angular trajectories, but not a significant difference in body CoM dynamics. The variance in quail leg trajectory suggests more complex factors in the most crouched species, such as nonlinearity of leg stiffness and/or greater use of rotational leg actuation. Nonetheless, quail do show increasing leg extension with obstacle height (Fig. 4), and posture-dependent actuation similar to that of other species (below).

**Fig. 5.**
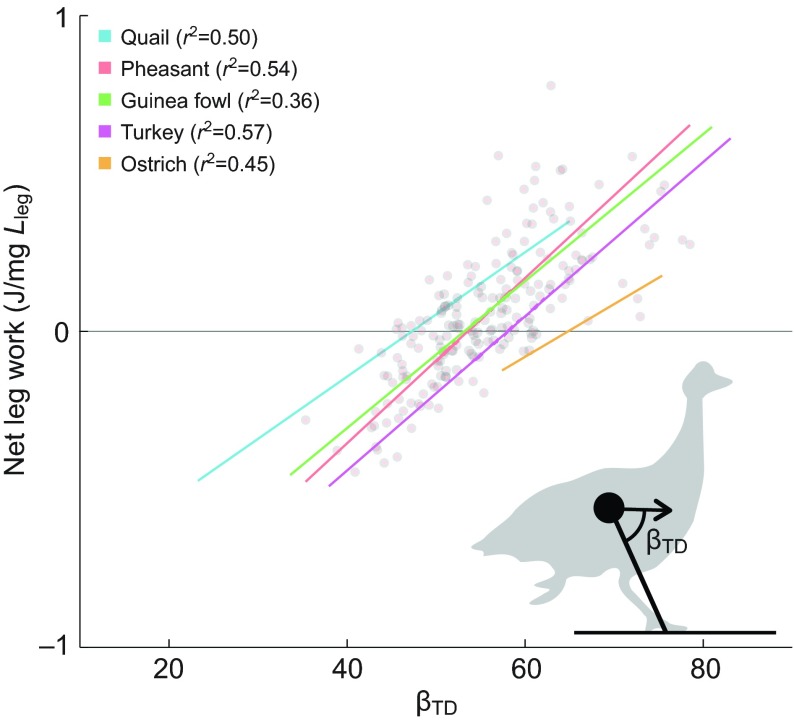
**Across species and terrains, we found a significant positive correlation between leg loading angle, β_TD_ (the angle between the leg and the body velocity at touchdown), and work done by the leg during stance.** Regression fits are shown for each species, with a representative data set shown for pheasants (all step types and all terrains).

Across species, we observed a consistent correlation between net limb work produced during stance and landing conditions, a pattern previously reported as ‘posture-dependent actuation’ (Birn-Jeffery and Daley, 2012; Daley and Biewener, 2011). We found a significant positive linear correlation between the leg loading angle (β_TD_, the angle between the leg and body velocity vector) and net limb work across species and body size (Fig. 5; *R*^2^ between 0.36 and 0.57). The slope of this relationship was remarkably similar across species, demonstrating a consistent pattern of posture-dependent leg actuation, which adjusts body mechanical energy on uneven terrain by inserting and removing energy depending on landing conditions. Thus, two key aspects of leg actuation patterns are consistent across species: (1) positive work actuation through leg extension in late stance, and (2) scaling of the magnitude of net work through posture-dependent actuation. Thus, we find that species spanning a 500-fold range in body mass employ similar leg actuation strategies for obstacle negotiation.

To investigate the implications of bird leg actuation patterns for economy of locomotion, we tested whether a model with minimum-work actuation could replicate the observed force and leg-length

**Fig. 6.**
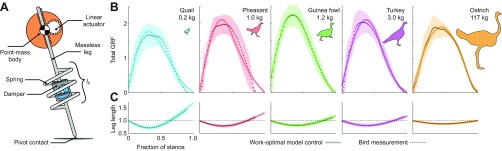
**Asymmetric force and leg length trajectories can be explained by minimal-work optimisation applied to a damped-spring-mass model with a linear actuator in series with the passive elements.** (A) Schematic of the model used to simulate running. (B) Comparison of bird GRFs in steady aerial running (mean ± s.d.) with force predicted by minimal-work optimisation. (C) Virtual leg length (distance between body CoM and foot point) for the work-optimal solution compared with the measured virtual leg length of each species (mean ± s.d.).

trajectories of running birds (Figs 6, 7). We found that stance force and leg dynamics closely match the predictions of a damped-spring-mass-leg model with minimal work applied through an actuator in series with the passive elements (Fig. 6). By fitting two parameters

**Fig. 7.**
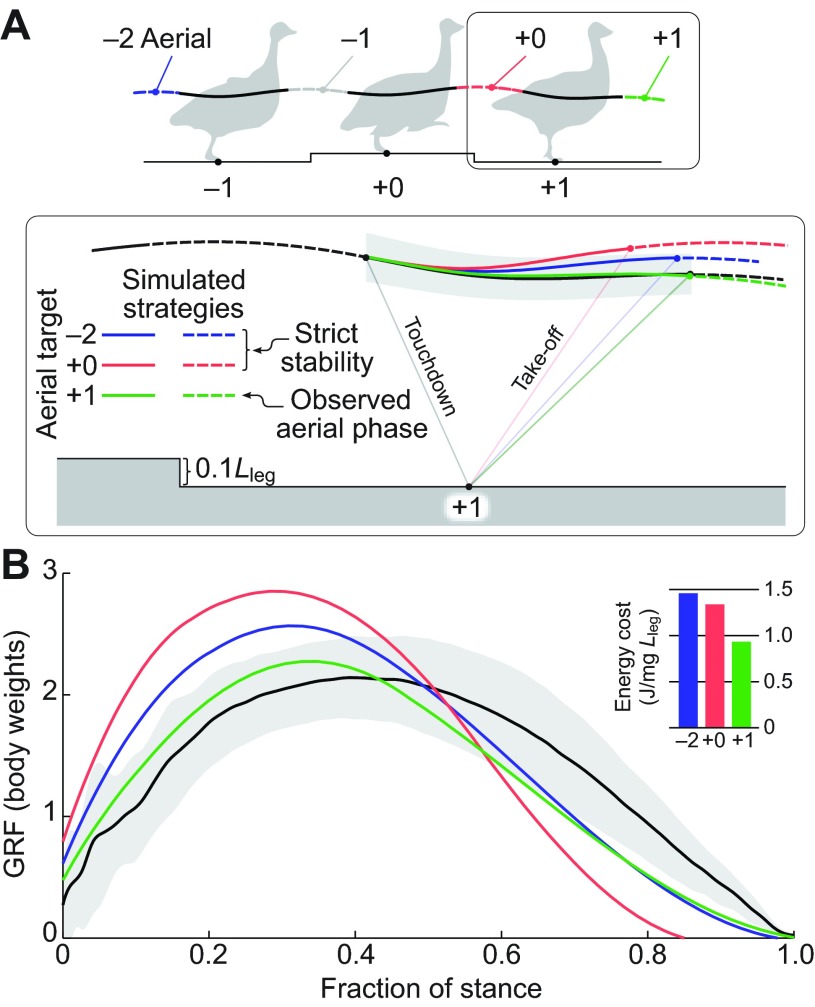
**Simulations predicting the force and energy consequences of targeting body stability during the obstacle dismount (0.1*L*_leg_) further suggest that birds target injury avoidance and economy rather than body stability.** We used simulation and trajectory optimisation methods identical to those used for level running (Fig. 6), with model parameters, including leg stiffness and damping, fixed to those for guinea fowl level running. (A) Comparison of the measured CoM trajectories (mean ± s.d.) and three simulated trajectories. The first two trajectories target alternative hypothetical interpretations of body stability, actuating with minimal work with the constraint to match the aerial phase of the undisturbed step −2 (blue) or the immediately preceding step +0 (red). The third trajectory (green) matches the experimentally observed step +1 aerial phase. (B) GRF for the simulated and measured (mean ± s.d.) strategies and their respective actuator work.

(leg stiffness and damping) and applying work-minimising optimisation, we found good fits to force and leg length trajectories across species for level terrain locomotion [mean squared error (MSE)=0.01–0.03, see supplementary material Figs S1–S3 and Table S7 for further model analysis]. All species exhibited similar goodness-of-fit between the model and the experimental data. The model predicts leg actuation in the second half of stance through net extension of the leg, similar to observed leg trajectories (Fig. 6C). Thus, work-optimal solutions derived from this model suggest that it is most economical to actuate the leg during the second half of stance.

Finally, we found that a model simulating body stability as a control target during the obstacle dismount (Fig. 7A) demonstrates that directly targeting a return to steady gait requires faster loading rates, higher peak forces and greater leg work than observed in the experimental data, even for the smallest 0.1*L*_leg_ obstacles (Fig. 7B). These simulations, along with the experimental data, suggest that birds do not directly target body stability as a control priority, but instead use unsteady body dynamics in a strategy prioritising economical energy management through minimal actuation, using posture-dependent actuation to negotiate obstacles with minimal fluctuations in leg posture and leg loading across several steps.

## DISCUSSION

### Obstacle negotiation strategies as a window into task-level control priorities

We examined running dynamics of cursorial birds spanning a 500-fold range in body mass as they negotiated a visible, single-step obstacle. Potential manoeuvres to negotiate this obstacle span a spectrum between two hypothetical extremes: ‘crouching’, which prioritises body stability, minimising fluctuations in body trajectory and external mechanical work, versus ‘vaulting’, which requires external mechanical work but minimises fluctuations in leg force–length dynamics. If birds directly targeted body stability as an active control priority, we would expect, at least for small obstacles, that they could use postural changes to avoid deviations in body trajectory from steady gait. Such behaviour has been observed in humans hopping and running on varying terrain (Ferris et al., 1999; Ferris et al., 1998; Grimmer et al., 2008; Moritz and Farley, 2003; Moritz and Farley, 2004; Moritz and Farley, 2006). The birds' strategy, while achieving general stability (they do not fall) is inconsistent with body stability as the direct target of active control. We find, instead, across species and terrain heights, that birds negotiate the obstacle using a consistent balance between ‘vaulting’ and ‘crouching’ that minimises fluctuations in leg posture and leg loading across multiple steps. We therefore reject the hypothesis that birds directly target stability of body CoM trajectory as a predominant control priority in uneven terrain.

The obstacle negotiation behaviour of birds is consistent with economical whole-body energy management as a key locomotor control priority. Posture-dependent actuation regulates the total mechanical energy of the body, through energy insertion and absorption (Fig. 5), and modelling studies have demonstrated that it has a stabilising effect on uneven terrain (Schmitt and Clark, 2009). Posture-dependent actuation also helps attenuate fluctuations in leg posture and loading, because it directly links leg force–length dynamics to work output. Altered leg posture and loading at touchdown elicit the actuation necessary to return the system towards the total mechanical energy of the nominal steady gait (Daley and Biewener, 2011). Additionally, posture-dependent work occurs in late stance (Daley and Biewener, 2011; Daley et al., 2009), coinciding with predictions of our minimum-work model (Fig. 6). Thus, birds may achieve general stability through economical energy management, via posture-dependent actuation, rather than through direct control of body CoM trajectory.

We found a striking similarity in the slope of posture-dependent actuation across species (Fig. 5). Posture-dependent actuation has also been previously observed in guinea fowl negotiating drops (Daley and Biewener, 2006; Daley et al., 2007), pheasants negotiating obstacles (Birn-Jeffery and Daley, 2012), and humans negotiating obstacles (Müller et al., 2012). This phenomenon may arise from shared features of vertebrate locomotor systems, including intrinsic musculoskeletal properties and reflex mechanisms. In vertebrate legged locomotion, stance phase muscle activity is determined through a combination of feedforward and feedback control, with the feedforward activation starting late swing, in anticipation of stance, because of significant neuromuscular delays (Daley and Biewener, 2011; Daley et al., 2009; Dietz et al., 1979; Donelan and Pearson, 2004; Engberg and Lundberg, 1969). Deviations between anticipated and actual leg loading lead to altered intrinsic dynamics and feedback-mediated changes in muscle force and work (Daley and Biewener, 2011; Daley et al., 2009). The observation of posture-dependent actuation in humans and several species of avian bipeds suggests that it may be a general feature of vertebrate legged locomotion.

Our reduced-order, minimum-work model of running further supports economical energy management as a key control priority of avian bipedal locomotion. The model successfully replicates the asymmetric force and leg-length trajectories of running birds (Figs 6, 7), and reveals that actuation through leg extension in late stance is economical for an intrinsically damped leg. Consistent with this, birds increased leg actuation in late stance when vaulting onto the obstacle (Fig. 4). However, the simple prismatic leg model does not encode postural gearing effects on muscle force, and therefore cannot predict the specific balance of ‘vaulting’ and ‘crouching’ onto obstacles used by running birds. The observed balance likely results from a trade-off between costs of external mechanical work (Minetti et al., 1994; Srinivasan and Ruina, 2006) and increases in muscle force costs associated with crouching (McMahon et al., 1987). Future studies could investigate this further by incorporating leg kinematics and postural costs into a leg model. Nonetheless, the current model does replicate key features of bird running, including the asymmetric force and leg length trajectories (Fig. 6) and obstacle dismounting behaviour (Fig. 7). The force asymmetry may be a universal feature of legged animal locomotion (Cavagna, 2006), but energy-conservative models, such as a spring-mass model, cannot reproduce it (Blickhan, 1989; McMahon and Cheng, 1990). Thus, our minimum-work actuation model provides a more accurate and explanatory reduced-order template of animal locomotion and supports economical energy management as key task-level priority governing leg control in running birds.

The observed obstacle negotiation strategy is also consistent with load regulation for injury avoidance as a key priority of leg control in running birds. Peak leg forces remained within 0.35 BW of level for obstacles up to 0.5*L*_leg_. Swing leg trajectory likely played a crucial role in controlling landing conditions to regulate leg loading. While the current study did not examine swing-leg dynamics in detail, previous studies have demonstrated the crucial role of swing-leg trajectory in determining landing conditions, and the coupling between swing and stance dynamics (Blum et al., 2014; Daley and Usherwood, 2010; Karssen et al., 2011; Vejdani et al., 2013). Birds use a swing-leg trajectory involving leg retraction in late swing (Birn-Jeffery and Daley, 2012; Daley and Biewener, 2006), with the specific retraction velocity tuned to target landing conditions that minimise fluctuations in leg loading in uneven terrain (Blum et al., 2014). Late-swing retraction velocity also determines the maximum terrain drop before the leg misses stance entirely, and is therefore crucial for avoiding falls (Blum et al., 2011; Daley and Usherwood, 2010). A swing control strategy that has been hypothesised in theory, but not observed in animals, is to optimise swing-leg trajectory to minimise deviations in body CoM trajectory, prioritising body stability (Blum et al., 2014; Ernst et al., 2012; Vejdani et al., 2013). However, this strategy can result in large peak leg forces – for example, increasing by +2–3 BW for a 0.4*L*_leg_ downward step (Blum et al., 2014; Vejdani et al., 2013). These forces could encroach dangerously towards safety factors of vertebrate bone, which are two to four times peak steady locomotor forces (Biewener, 1990). Even submaximal increases in force can lead to micro-damage and repetitive loading injury if insufficient repair occurs between bouts (Guo et al., 1994; Verheyen et al., 2006). Birds consistently preserve similar peak forces across uneven terrain, both in this and in previous studies (Birn-Jeffery and Daley, 2012; Blum et al., 2014; Daley and Biewener, 2006), suggesting injury avoidance as a key control priority.

Regulation of peak forces on uneven terrain also further supports economy as a priority, because minimising forces reduces energy expenditure. The metabolic energy cost of locomotion depends strongly on both muscle work and force (Kram and Taylor, 1990; Minetti et al., 1994; Srinivasan, 2010; Srinivasan and Ruina, 2006). The cost of external mechanical work by vaulting onto the obstacle may be offset by avoiding excessively crouched postures on the obstacle, which would increase muscle force costs, because of changes in gearing (Biewener, 1989; McMahon et al., 1987). Thus, overall, our experimental and modelling evidence suggest that both economy and injury avoidance are crucial and closely coupled task-level priorities governing leg control in running animals.

### Does body size influence non-steady locomotor control priorities?

Body size affects morphology (Christiansen, 1999; Doube et al., 2012; Kilbourne and Hoffman, 2013), locomotor performance (Hoyt et al., 2000; Iriarte-Díaz, 2002; Jackson and Dial, 2011; Tobalske and Dial, 2000; Walter and Carrier, 2002) and physiology (Biewener, 1989; Hoyt and Taylor, 1981; More et al., 2010; Nagy, 1987; Taylor et al., 1982). As animals increase in size, they tend towards more upright, straight-legged posture, which reduces the muscle stresses required to support body weight (Biewener, 1989; Biewener, 2005). Here, we present findings that suggest that these scaling trends in musculoskeletal structure do not substantially influence obstacle negotiation strategies. Birds spanning a 500-fold range in body mass used consistent obstacle negotiation manoeuvres and similar leg actuation patterns. The observed manoeuvres suggest economical energy management and injury avoidance as key priorities, irrespective of body size and leg posture. In the wild, injuries can result in predation, and food energy resources are often limited, thus, injury avoidance and economy are likely to be important factors in fitness.

Understanding how body size influences bipedal locomotion can provide insight into the co-evolution of behaviour and morphology among living and extinct animals, allowing us to better reconstruct the behaviour of extinct animals from fossil evidence (Hutchinson and Garcia, 2002). Birds share numerous features of leg morphology with non-avian theropod dinosaurs, such as *Velociraptor*, a dromaeosaur and the bird-like troodontids (Padian and Chiappe, 1998). Although the largest theropods may not have been fast runners (Hutchinson and Garcia, 2002), evolution of striding bipedalism among theropod dinosaurs and their bird descendants (Farlow et al., 2000) may reflect selection for robust and economic locomotion in uneven terrain. Bipeds tend to have longer leg lengths compared with quadrupeds of similar body size (Roberts et al., 1998b), which may allow them to negotiate larger obstacles with minimal external work and postural change. We suggest that unified leg control among ground birds may reflect a shared heritage of bipedal agility in the lineage of theropod dinosaurs and their bird descendants.

Birds do not exhibit a shift in obstacle negotiation strategy with body size, and we suspect that similar examples may be found in other animal orders. However, the specific strategies used may vary across clades. Animals with substantially different locomotor mode and leg number (e.g. quadrupeds, hexapods) may use different strategies to negotiate obstacles because increased leg number allows greater intrinsic stability and a larger range of behavioural options (Olberding et al., 2012; Spagna et al., 2007; Sponberg and Full, 2008; Watson et al., 2002). Additionally, the animals used in this study are large relative to the body size range among extant animals (e.g. insects). Small animals do not suffer from long physiological delays (More et al., 2010), and may use feedback mechanisms on a relatively shorter time scale. These factors may result in different specific strategies among different clades. Nonetheless, previous obstacle negotiation studies in smaller animals have also observed anticipatory strategies involving substantial changes in body dynamics and leg posture (Kohlsdorf and Biewener, 2006; Sato et al., 2012; Watson et al., 2002). The present study suggests that body size alone does not necessarily lead to a shift in control priorities and obstacle negotiation strategy within a group of animals with similar locomotor style (e.g. striding bipeds). Future work should address whether similar control priorities are observed among legged animals in different clades.

### Potential implications for control of legged robots

In engineered systems, such as robots and prosthetics, using body stability as a direct control priority is commonplace because it maintains the system within known dynamics and control authority (Westervelt et al., 2007). This approach takes many forms, e.g. locally stabilising a nominal gait with feedback (Bhounsule et al., 2012), designing gaits that result in smaller gait deviations when perturbed (Dai and Tedrake, 2012), planning rapid returns to a nominal gait (Erez et al., 2012), or rejecting perturbations entirely (Ernst et al., 2012). Our results suggest a different approach, embracing the looser definition of stability as ‘fall avoidance’,

**Fig. 8.**
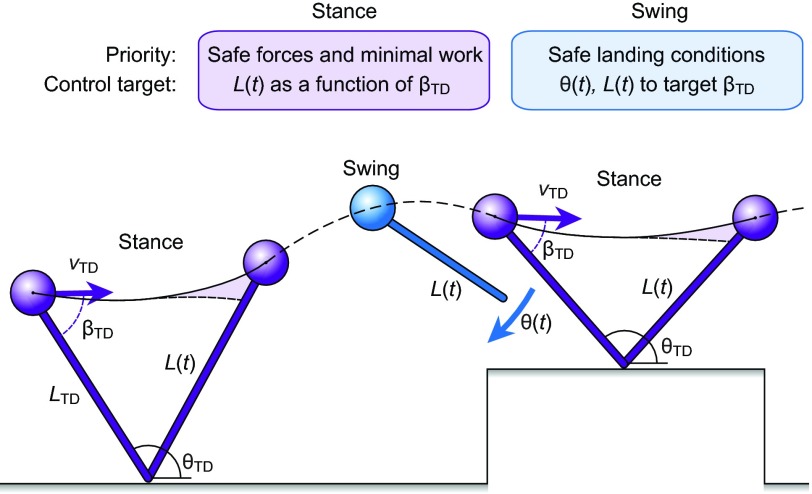
**Schematic illustration of the hypothesised task-level priorities and leg control targets of running birds, highlighting principles that have emerged from this and other recent studies (see Discussion).** Birds may achieve safe leg forces and economical step-to-step energy management in uneven terrain by controlling swing leg trajectory to target safe landing conditions (Blum et al., 2014), and applying cost-minimising actuation during stance, through leg extension in the latter half of stance (Fig. 6). Posture-dependent actuation tightly couples swing and stance dynamics (Fig. 5), and allows economical energy management while also attenuating fluctuations in leg posture and loading.

consistent with the mathematical concept of ‘metastability’, which has been recently extended to the analysis and control of legged systems (Byl and Tedrake, 2009).

### Conclusions

The findings here, in the context of previous studies, support a unified model and set of task-level control priorities for robust and economical bipedal locomotion, schematically illustrated in Fig. 8. The model is a damped, spring-mass system with an actuator in series with the passive elements (Fig. 6A), and the task-level control priorities are: (1) ensuring leg–ground contact to avoid falls, while (2) regulating peak force and leg posture for economy and leg safety and (3) applying minimal work actuation to manage the body total mechanical energy. The observed convergence toward steady gaits may be a consequence of these priorities, not a direct objective (not direct targeting of a nominal desired trajectory). Our findings refute stability of body CoM trajectory, per se, as a direct priority of control in running birds. We suggest that for bipedal robots and prosthetics to match the robust, agile and economic locomotion of animals, control approaches must embrace a more relaxed notion of stability, optimising dynamics based on key task-level priorities without encoding an explicit preference for a steady gait.

## MATERIALS AND METHODS

### Experimental protocol

We collected both kinematic (body and leg motion) and kinetic (GRF) data for five cursorial ground bird species spanning a 500-fold body mass range: northern bobwhite quail (*Colinus virginianus, N*=6, mass 0.22±0.02 kg), common pheasant (*Phasianus colchicus, N*=4, mass 1.03±0.21 kg), helmeted guinea fowl (*Numida meleagris, N*=5, mass 1.24±0.30 kg), wild North American turkey (*Meleagris gallopavo, N*=6, mass 3.0±0.3 kg) and ostrich (*Struthio camelus, N*=4, mass 116.8±6.1 kg). All birds, except the ostriches, had their primary wing feathers clipped to prevent flight. The ostrich's wings obscured leg and body markers so the distal end of each wing was wrapped in Vetwrap™ to restrict wing fanning. This did not appear to have adverse effects on medio-lateral stability. The Royal Veterinary College Ethics and Welfare Committee approved all procedures under the project protocol title ‘Kinematics and kinetics in birds running over uneven terrain’. The protocols did not require any invasive or surgical procedures.

In this study, birds ran over terrain with a single-step visible obstacle, with ample distance, time and practise runs to anticipate the obstruction, allowing them to optimise behavioural strategy based on task-level priorities (e.g. stability, minimal work, safe forces, etc.). The obstacle required a single step upon the obstacle before stepping back down (not ‘hurdling’). This visible, single-step perturbation contrasts with a persistent terrain change, for which recovery to the unperturbed gait might be unreasonable, or an unexpected perturbation, which reveals intrinsic stability mechanisms but does not necessarily reflect an optimised strategy.

Level terrain running served as a control for each species. All birds except ostriches ran over obstacle heights scaled between 0.1 and 0.5 nominal leg length (*L*_leg_). A substantial scaling effect on leg posture has been reported by Gatesy and Biewener (Gatesy and Biewener, 1991) based on a ‘posture index’ equal to the ratio of hip height to the sum of the hindlimb segment lengths. Our posture index values ranged from 0.36 in the quail to 0.74 in the ostrich when measured at mid-stance during moderate-speed level running (Fig. 3). To compare intrinsic stability and control strategies across species, we wanted to compare behaviour in appropriately scaled terrain conditions. Yet, the co-variance between leg posture and body mass in animals makes it challenging to tease apart their respective effects. Scaling the obstacles based on a bird's hip height would be problematic for two reasons: (1) birds do not have a single ‘true’ hip height that can be definitively measured, because standing posture varies considerably from ‘mid-stance’ running posture, and (2) because of the substantially crouched posture of small birds, scaling of obstacles based on hip height would amplify the apparent stability of smaller animals for their body mass. Consequently, we reasoned that the fairest comparison between animals of differing body mass should be based on scaling of obstacle heights relative to an isometrically scaled leg length reference value. We therefore calculated a nominal leg length based on body mass and assumptions of geometric similarity, without the confounding effect of leg posture, using the following formula:
(1)
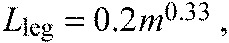

where *L*_leg_ is leg length and *m* is body mass, and the coefficient 0.2 was selected to obtain a leg length proportional to an intermediate posture (approximately that of a turkey). This obstacle scaling choice means that, relative to mid-stance hip height, obstacles were larger for the more crouched species. We feel this is a fair, conservative test for the hypothesis that crouched postures reflect increased priority for stability on rough terrain.

Each obstacle was placed in the mid-section of a runway with ample length at either end to allow acceleration to a steady speed before encountering it. The obstacle spanned the medio-lateral width of the runway, so it was not possible to manoeuvre around it. To minimise experiment complexity, we restricted obstacle heights to those the birds could run over (continuous striding with positive forward velocity), to avoid categorical shifts in behaviour to jumping. The birds were encouraged to run back and forth across the runway by locating dark resting boxes at either end (for smaller species), or a pen at one end containing a few members of the flock (for the ostriches).

The ostriches were restricted to 0.1*L*_leg_ obstacles because of safety considerations for the birds and handlers. However, comparison of 0.1*L*_leg_ obstacle negotiation across species is the most appropriate for scaling comparisons because the potential for different strategies is most pronounced for small obstacles. A shift away from steady gait is clearly required once obstacles reach heights that challenge stance posture or swing foot clearance limits. However, for small obstacles, use of postural strategies to achieve a steady gait is a plausible option that could minimise external mechanical work (Fig. 1). Comparing species negotiating a similarly scaled obstacle allows us to evaluate the extent to which they employ leg postural strategies (‘crouching’) to attenuate deviations in body CoM trajectory in uneven terrain, a strategy that suggests body stability as a control priority.

### Data collection and processing

We collected GRF data at 500 Hz from force plates embedded in the runway. Force data were pre-filtered using a low-pass filter of 100 Hz. For all birds excluding the quail, the runway contained six Kistler force plates (0.6×0.9 m; model 9287B, Hook, Hampshire, UK). Because of the lack of resolution in the model 9287B force plates for small animals, we created a different runway for the quail, containing two ‘Squirrel’ Kistler plates (0.12×0.2 m; model Z17097, Hook, Hampshire, UK), but the experimental procedures and data collection protocol were otherwise identical.

We collected kinematic data at 250 Hz from markers placed cranially and caudally on the birds' back and on the feet located at the tarsometatarsalphalangeal joint and digit III. The back markers were averaged for an initial estimate of the body CoM velocity and position. The foot markers were averaged to estimate foot position and calculate the effective leg length and angle. For all birds, except the ostriches, the kinematics were recorded using eight to 12 Qualisys cameras (Gothenburg, Sweden) placed evenly around the field of view. Because of difficulties with maintaining Qualisys markers on the ostriches, the ostrich data were collected using high-speed video (HSV) cameras (AOS Technologies AG, Dättwil, Switzerland). Paper markers were placed on the same landmarks. HSV was collected using two lateral view cameras on either side of the runway. Sagittal plane 2D data points were digitised in the DLTdv5 code (Hedrick, 2008). Kinematic recording devices were triggered synchronously with the force plates. For simplicity, all data analyses were restricted to the sagittal plane, considering only the vertical and fore–aft dynamics in both the experimental and modelling analyses.

Step types across the runway were identified with respect to the obstacles, where the ‘on’ obstacle step was defined as ‘step 0’. We collected at least six trials per condition per individual within each species. We included trials in which the bird ran in a straight line and appeared steady to the human eye in the initial approach to the centre of the runway. In post-processing, we selected steady approach trials by restricting the analysis to trials in which the fore–aft impulse of ‘step −2’ (two steps before the centre) was within ±1 s.d. of the level data distribution for each species. A net zero impulse corresponds to perfectly steady forward locomotion. Across species, this fore–aft impulse criterion corresponded to a maximum 10% change in forward speed. This criterion minimises the variance due to acceleration in the initial approach before encountering the obstacle, but does not restrict non-steady strategies for obstacle negotiation. The data were segmented into step cycles for all subsequent analysis. Once only steady trials had been selected, the numbers of level trials included in the statistical analysis were: quail, 96; pheasant, 33; guinea fowl, 91; turkey, 255; and ostrich, 39. And for the obstacle trials: quail, 362; pheasant, 163; guinea fowl, 240; turkey, 333; and ostrich, 25.

We twice-integrated forces to obtain body CoM motion, using initial conditions obtained following previously described methods (Birn-Jeffery and Daley, 2012). We then calculated mechanical energies, peak forces and leg posture over the step cycle, defined as touchdown of one foot to touchdown of the contralateral foot. In obstacle terrains, average trajectories were calculated for steps approaching the obstacle (step −1), on the obstacle (step 0) and dismounting the obstacle (step +1). As a control reference, average trajectories and 95% confidence intervals were generated from level terrain data for each species.

### Statistical analyses

All statistical tests were run following checks for normality and were completed using MATLAB R2012a with the Statistics toolbox. To allow comparisons across species and minimise variance due to within-species individual size differences, all variables in the analysis were normalised to dimensionless quantities based on body mass, gravity and nominal leg length *L*_leg_ (Birn-Jeffery and Daley, 2012; McMahon and Cheng, 1990). Average forward speeds differed among species; in particular, the quail and ostrich ran at lower average speeds than the other species. Therefore, to control for the effects of speed in the comparisons across species, we restricted the data to normalised velocities between 0.75 and 2.00 and included speed as a covariate in the statistical analyses.

For the statistical analysis of obstacle negotiation dynamics, we took the difference between the obstacle terrain values and the level terrain mean value, thus measuring the deviation from steady gait. This means that any statistical differences among species reflect differences related to obstacle negotiation, not differences in steady-state gait.

For the species with multiple obstacle height conditions (galliforms), we ran an ANOVA with the factors obstacle height, step type and their interaction, species and the interaction term between species and step type, speed as a continuous covariate and an interaction term between speed and species. If the main effects were found to be significant for a specific variable, we ran *post hoc* pairwise comparisons with a sequential Bonferroni correction (Holm, 1979; Rice, 1989) (see supplementary material Tables S4–S6). The pairwise comparisons (supplementary material Tables S5–S6) were completed using MATLAB function ‘multcompare’.

We separately analysed the 0.1*L*_leg_ obstacle data across all species including ostrich, using an ANOVA with step type as a fixed effect, species as a random factor, the interaction between step type and species, speed as a continuous covariate and an interaction term between speed and species. *Post hoc* pairwise comparisons with sequential Bonferroni corrections were completed using ‘multcompare’ if the main effects were found to be significant (see supplementary material Tables S1–S3).

For the regression analyses shown in Fig. 4, we used reduced major axis least-squares regression to test for a linear relationship between β_TD_ and net leg work. A single regression was fit for all data from each species, including all steps in level and obstacle terrain conditions. Outliers were removed from the regression for values greater than 3 s.d. from the mean.

### Modelling

We used a simple, reduced-order dynamical model (Fig. 6A) to quantitatively analyse the trajectories of bird locomotion, particularly, a non-conservative variant of the spring-loaded inverted pendulum (SLIP) model. The SLIP model of running has long been used to model the energy exchange, CoM trajectories and GRF of biological (Blickhan, 1989; Daley and Biewener, 2006; McMahon and Cheng, 1990) and robotic (Altendorfer et al., 2001) runners. This model features a lumped mass body, a massless leg, a frictionless pivot at the point of ground contact, and a linear leg spring that connects the body and ground. The total GRF exerted by the leg in the SLIP model has a characteristically symmetric half-sine shape as the spring stores and releases energy conservatively during stance.

To account for GRF asymmetry, we took our model out of the energy-conservative regime by adding a damper to simulate realistic energy losses, and an actuator in series with the spring and damper to replace the lost energy. We modelled the inherent leg dissipation as a linear damper, acting in parallel with the linear leg spring. To reinsert energy into the system, we include an axial actuator in series with the spring, analogous to a muscle in series with a springy tendon. This model decouples force and posture, such that the model allows arbitrary force–length trajectories (supplementary material Fig. S1). We did not place any inherent limitations on the motion of the actuator, e.g. acceleration or length limits, although the trajectory optimiser ultimately found solutions that did not require unrealistic accelerations. This linear actuator can only act in the axial direction. The equations of motion for the actuated model are as follows:
(2)
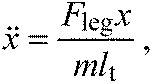

(3)
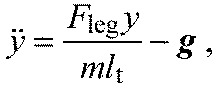

where *F*_leg_ is axial leg force, *l*_t_ is total leg length and *x, y* are the Cartesian coordinates of the body mass relative to a foot-point origin, with unsigned gravitational acceleration (***g***), body mass (*m*) and:
(4)
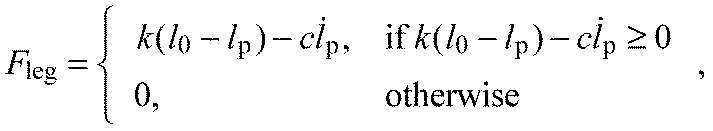

(5)
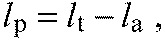

(6)
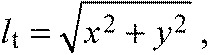

where *l*_a_ is the actuated leg length, *l*_p_ is the passive leg length and *i*_p_ is the velocity of the passive leg length (Fig. 6A). Parameters *k* and *c* are the model spring stiffness and damping coefficient, respectively.

### Processing measurements into model boundary conditions

Our data processing methodology took averaged GRF and CoM trajectories for each species and converted them into parameters and boundary conditions for the actuated model. The experimental data used for the modelling were restricted to aerial running trials (not walking or grounded running). For the model to be useful, if we simulate a point mass with the empirically measured touchdown (TD) conditions and ‘play back’ the measured bird GRF, we should see an integrated CoM trajectory that matches the mean measured bird CoM trajectory. However, point-mass locomotion models are inherently sensitive to TD conditions, meaning that even small errors of this single time-point measurement can lead to significant deviations between the mean-measured bird CoM trajectory and the ‘point-mass playback’ simulation (in essence, a dynamical disagreement).

Using optimisation, we adjust the four TD state variables (leg length, leg angle, velocity magnitude and velocity angle) and flight phase duration to minimise the discrepancy. We minimised the Euclidean distance between measured CoM trajectory and the point-mass playback, adjusting them by no more than 1 s.d. from the mean, while traveling the same horizontal distance during the full step cycle. Sequential Quadratic Programming (SQP, as implemented by MATLAB's fmincon) was used for optimisation and all equations of motion were integrated using MATLAB's ode45 (tolerance 2.23×10^−14^).

Final state targets for the trajectory optimisation are constrained to intersect the state trajectory of the subsequent aerial phase (velocity magnitude, velocity angle, vertical position, and not the horizontal position). While targeting the precise measured take-off state for the measured stance phase would seem an obvious choice, targeting a single exact state is often unnecessarily constraining to the optimisation problem. Instead, targeting the aerial phase permits more solutions with slightly different take-off leg lengths and distances traversed, allowing the optimiser more freedom to select the most energy-efficient option while still achieving the observed gait dynamics. We argue that the close trajectory matches to measured data in spite of the greater optimiser freedom strengthens the case for the validity of the model.

### Trajectory optimisation

To facilitate energy-optimal control, we numerically solved for the work-optimal GRF for a simplified actuated model (Fig. 6A) using trajectory optimisation. For each species, this model was given average measured bird mass and landing conditions from processed experimental data. The optimisation found the actuator's leg-extension trajectory which minimises the net unsigned work, a simple proxy for metabolic cost (Srinivasan and Ruina, 2006):
(7)
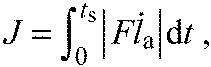

where *J* is the value of the objective function, *F* is the leg force, *i*_a_ is the velocity of the actuator thrust and *t*_s_ is the duration of the stance phase. We use smoothed approximation of the absolute value function, as used in prior optimisation studies (Srinivasan and Ruina, 2006), 
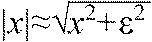
, where ε is small (0.001). We also imposed hard equality constraints on the optimiser to find solutions that satisfied the boundary conditions calculated by the data processing, allowing for differences in total distance travelled, step length and speed to avoid overly constraining trajectories.

Using a multiple-shooting formulation (Bock and Plitt, 1984) allowed for more reliable trajectory solving with a wider array of initial guesses, discretising the input tape into a 20-segment piece-wise differentiable curve (much finer resolutions did not yield any significant differences). We solved the optimization problem using an SQP solver (implemented using MATLAB's fmincon) and different initial guesses were spot-checked, never revealing different solutions of any significance.

### Parameter search

While all other model parameters were experimentally measured, the parameters *k* and *c* must be fitted for the birds. To fit these two parameters, the trajectory optimisation was looped inside a gridded search, producing a two-dimensional table of work-optimal trajectories for a range of parameter values {*k*_norm_=[7:0.5:17] and *c*_norm_=[0.0:0.05:0.7], where *k*_norm_ is the normalised spring stiffness (*kL*_leg_/*m****g***) and *c*_norm_ is the normalised damping coefficient (
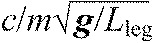
)}. We then selected the parameters for each species for which the work-optimal solution provided the closest fit to the mean measured GRF, as measured via MSE. The result was a fitted work-optimal prediction for the steady running of each species (Fig. 6B). Note that because the set of solutions were all work optimal for their respective parameter values, before selecting the best fit to data, there was no guarantee of a good match between model and data (see supplementary material Figs S1–S3). Therefore, the modelling approach could have failed to fit the data, potentially refuting the work-minimising hypothesis.

Searches for the best-fitting parameters, *k* and *c*, can be visualised as a fitting landscape (see supplementary material Fig. S3). Valleys in this surface reflect better quality fits, as defined by MSE between predicted and measured GRF trajectory. While some regions of this fitting landscape clearly performed better than others, there was often a large set of solutions that performed similarly well. Among these solutions, we eliminated a subset that performed actuator work at the instant of touchdown, because this immediate (and typically brief) period of work was associated with poorly matched parameter/boundary condition combinations. More details on the parameters of these best-fit trajectories are provided in supplementary material Table S7.

## Supplementary Material

Supplementary Material
