# Supplementary material for: Don't break a leg: running birds from quail to ostrich prioritise leg safety and economy on uneven terrain
Source: J Exp Biol. 2014 Nov 1;217(21):3786–96. doi: 10.1242/jeb.102640 (PMC4213177; doi:10.1242/jeb.102640)
Supplement: Supplementary Material [file supp_217_21_3786__index.html]

Don't break a leg: running birds from quail to ostrich prioritise leg safety and economy on uneven terrain — Supplementary Material 

# Don't break a leg: running birds from quail to ostrich prioritise leg safety and economy on uneven terrain

## JEB102640 Supplementary Material

**Files in this Data Supplement:**

- **Supplementary Material**
