# Supplementary material for: Don't break a leg: running birds from quail to ostrich prioritise leg safety and economy on uneven terrain
Source: J Exp Biol. 2014 Nov 1;217(21):3786–96. doi: 10.1242/jeb.102640 (PMC4213177; doi:10.1242/jeb.102640)
Supplement: Supplementary Material [file supp_217.21.3786_JEB102640.pdf]

Supplementary Figures S1-S3 and Table S7: Model analysis and results

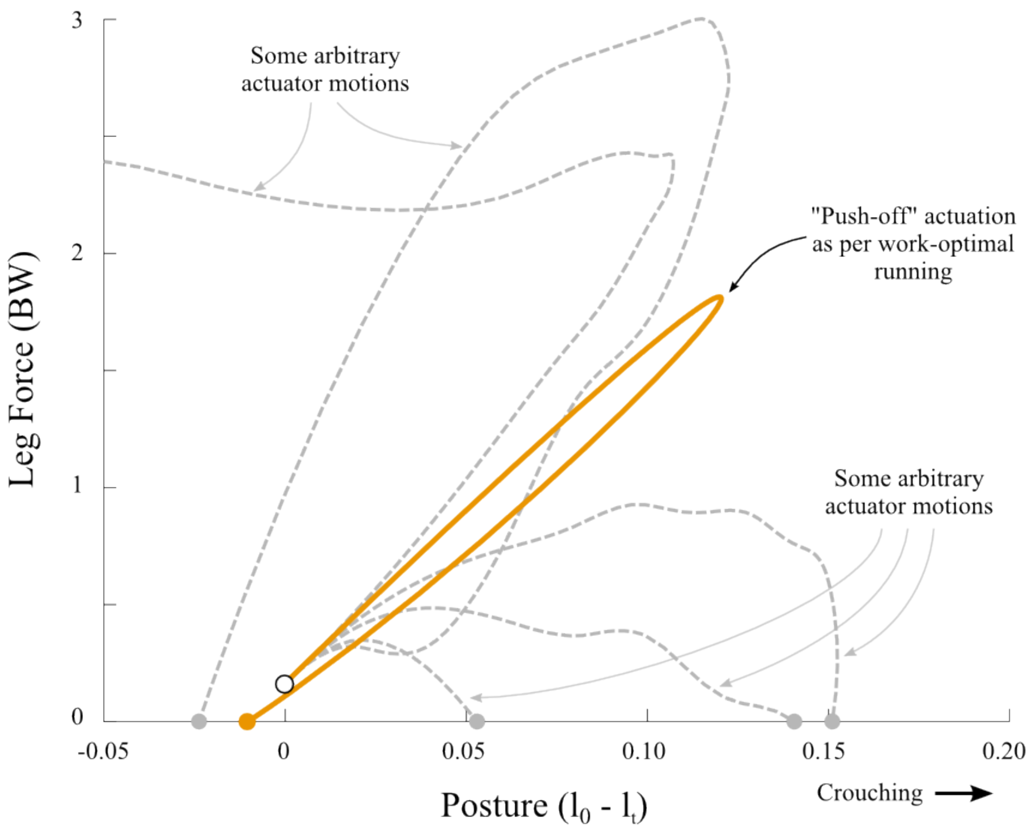

Figure S1. Force-posture relations for the actuated spring-mass-damper model with various and arbitrary actuator motions. Here, the model parameters and touch-down conditions have been held constant, and arbitrary actuator motions applied. This demonstrates a wide range of possible force-length relations with the mathematical model. The arrangement of the actuator in series with the spring and damper decouples posture from force, allowing for forces that deviate significantly from a Hooke's law relation. The specific force-length trajectory of the simulation results arises from minimal-work optimisation.

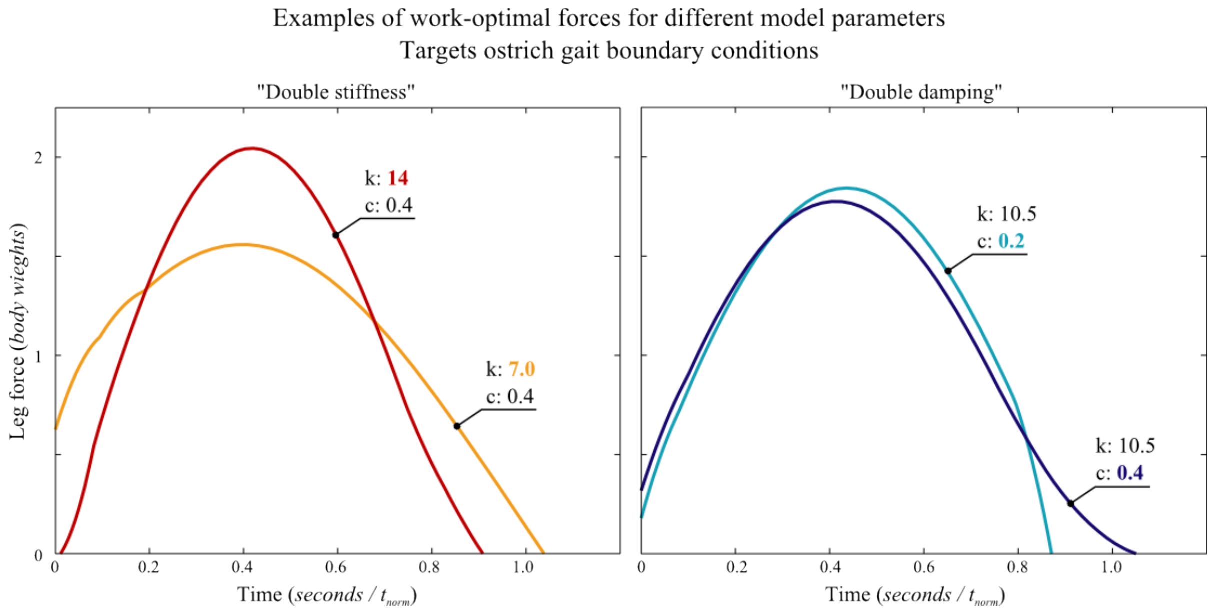

Figure S2. Example work-optimal solutions for the mathematical model satisfying the level-running ostrich gait boundary conditions (touchdown conditions of current and subsequent step). By changing either the model stiffness or damping coefficient by a factor of two, different work-optimal solutions emerge from the control optimization, which all satisfy the boundary conditions (i.e. the problem is not over-constrained). The modelling methods allow for freedom in take-off conditions, such that the model solutions could yield longer or shorter flight phases that satisfy the touch-down conditions for the subsequent step. Thus, the modelling approach can yield solutions with gait parameters and GRFs that deviate substantially from observed data. To make choice of stiffness and damping parameters non-arbitrary, we choose the model parameters for which a work-optimal control matched the data best (Fig. S3). However, the set of solutions from which these parameters were chosen (e.g., Fig. S3) were all work-optimal for their respective parameter values, and were not constrained to fit the bird data. Consequently, the modelling approach could have failed to fit the data, potentially refuting the work-minimising hypotheses.

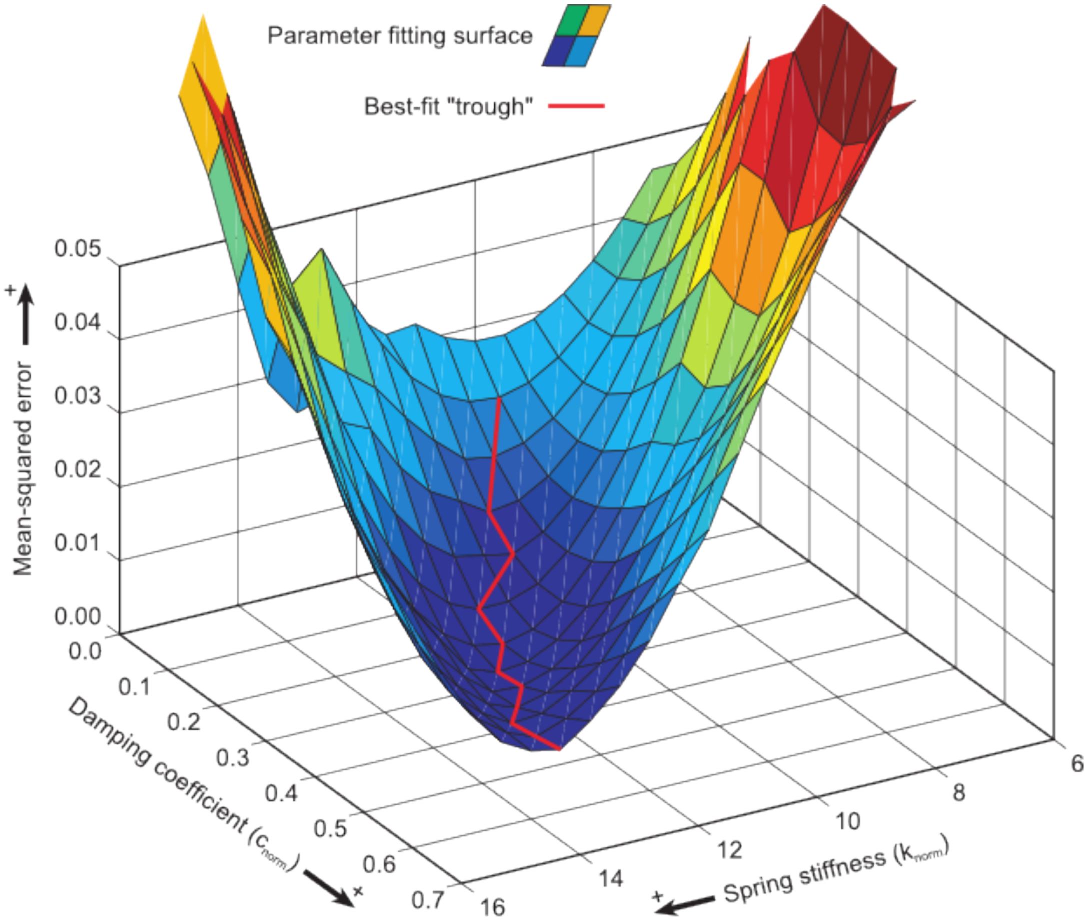

Figure S3: **A typical example of a parameter-fitting surface for the reduced order model of avian running:** The results of our search for the best fitting parameters to the simple model with minimal actuation (Fig. 6A), visualised as a fitting landscape. All solutions shown on the surface are work-optimal for their respective parameter values. In this example, computed using ostrich data, the surface shows a characteristic 'trough' of parameter fits that emerge when searching for k_{norm} and c_{norm} that best fit bird data. The red 'trough' line connects the best fits for each value of c_{norm}. Parameters are normalised as described Table S7, and mean-squared error is computed between model and mean-measured GRF. While some regions of this fitting landscape clearly performed better than others, there was often a large set of solutions that performed similarly well. Given the non-unique nature of the parameter fits, we do not make scientific claims about the functional significance of the fit set of parameters. Nonetheless, we did find a relatively narrow range of damping ratios (a standard measure of decay in oscillating systems) resulting in fits consistent with bird running data (Table S7). We report this as a successful result for the general model, which yielded good match between bird and model GRF, given a two-parameter fit (MSE: quail: 0.0103, pheasant: 0.0280, guinea fowl: 0.0032, turkey: 0.0086, ostrich: 0.0063, calculated by force error normalised to body weight).

Supplementary Tables S1-S6: Statistical results from experimental data

Dependent Variable	Step Type	Species	Species X StepType
**θ_{TD}**	**20.60**	**3.47**	**4.49**
**H_{TD}**	**41.85**	0.07	**2.77**
**α_{TO}**	**15.59**	1.69	**4.60**
**ΔE_P**	**31.25**	0.27	**3.55**
**ΔE_K**	**7.51**	0.78	**3.24**
**ΔE_{CoM}**	**8.64**	0.42	**2.97**
**F_{max}**	**5.06**	**6.07**	**2.69**

*Bolding indicates a statistically significant result

Table S1: ANOVA F-statistic results for 5 species, including ostriches, testing for effects of step type and species in $0.1L_{leg}$ obstacle terrain (see Methods). Degrees of freedom are as follows: step type = 3; species = 4; species x step type = 12; α_{TO} total = 743; all other variables total = 790. The F-statistic for the effect of step type on leg posture (θ_{TD}, H_{TD}) and change in potential energy (ΔE_p), which are most indicative of obstacle negotiation strategy, are much larger than the corresponding F-statistics for species (F < 1) and species x step type (F < 5). This reflects a uniform obstacle negotiation strategy across species the species studied here (see posthoc comparisons in Tables S2-S3 for further detail). All species used a consistent balance of 'vaulting' and 'crouching' strategies (Figs, 1 and 2).

Step 1	Step 2	θ_{TD} (degrees)	H_{TD}	α_{TO} (degrees)	ΔE_P	ΔE_K	ΔE_{CoM}	F_{max}
Level	Step -1	-0.80	0.015	**2.28**	**0.041**	**0.053**	**0.093**	0.01
Level	Step 0	**2.57**	**-0.044**	-0.36	0.015	-0.005	0.010	**-0.09**
Level	Step 1	**-2.41**	**0.056**	**-2.27**	**-0.051**	**0.065**	0.014	0.06
Step -1	Step 0	**3.37**	**-0.059**	**-2.63**	**-0.026**	**-0.057**	**-0.084**	-0.10
Step -1	Step 1	-1.61	**0.041**	**-4.55**	**-0.092**	0.013	**-0.079**	0.05
Step 0	Step 1	**-4.98**	**0.099**	**-1.91**	**-0.066**	**0.070**	0.005	**0.15**

*Bolding indicates significant difference based on Bonferroni threshold of 0.0083, for 6 possible step type pairwise comparisons within level and 0.1 L_{leg} obstacle height.

Table S2: Post hoc results on the ANOVA using pairwise mean differences between step types (column 2 - column 1), in normalised units.

Species 1	Species 2	θ_{TD} (degrees)	H_{TD}	α_{TO} (degrees)	ΔE_P	ΔE_K	ΔE_{CoM}	F_{max}
**Step -1**								
Quail	Pheasant	-6.89	-0.020	-3.73	0.016	0.117	0.133	**-0.51**
Quail	Guinea fowl	-5.26	-0.294	-1.05	0.021	0.021	0.042	-0.22
Quail	Turkey	-4.34	-0.037	-2.75	-0.025	0.009	-0.017	-0.14
Quail	Ostrich	-3.48	**-0.126**	0.36	0.027	0.030	0.056	-0.08
Pheasant	Guinea fowl	1.63	-0.010	2.68	0.005	-0.096	-0.092	0.30
Pheasant	Turkey	2.55	-0.018	-0.98	-0.042	-0.109	-0.150	**0.38**
Pheasant	Ostrich	3.41	-0.107	4.10	0.010	-0.087	-0.077	0.43
Guinea fowl	Turkey	0.91	-0.008	-1.70	-0.046	-0.012	-0.058	0.08
Guinea fowl	Ostrich	1.77	-0.097	1.41	0.006	0.009	0.015	0.14
Turkey	Ostrich	0.86	-0.089	3.12	0.052	0.021	0.073	0.06
**Step 0**								
Quail	Pheasant	-4.81	-0.032	1.71	0.014	0.159	0.174	-0.35
Quail	Guinea fowl	-2.95	-0.029	**4.57**	0.045	0.095	**0.141**	-0.05
Quail	Turkey	-3.36	-0.014	**3.73**	0.013	-0.030	-0.017	0.09
Quail	Ostrich	-1.47	-0.058	-0.36	0.013	0.061	0.074	-0.22
Pheasant	Guinea fowl	1.86	0.004	2.85	0.031	-0.064	-0.033	0.30
Pheasant	Turkey	1.45	0.019	2.01	-0.002	-0.189	-0.191	**0.45**
Pheasant	Ostrich	3.34	-0.025	-2.08	-0.001	-0.098	-0.099	0.13
Guinea fowl	Turkey	-0.41	0.015	-0.84	-0.033	**-0.125**	**-0.158**	0.14
Guinea fowl	Ostrich	1.48	-0.029	-4.93	-0.032	-0.034	-0.067	-0.16
Turkey	Ostrich	1.89	-0.044	-4.09	0.000	0.091	0.091	-0.31
**Step +1**								
Quail	Pheasant	-6.46	0.027	-3.99	-0.067	0.123	0.056	-0.12
Quail	Guinea fowl	**-7.77**	0.024	-1.53	-0.038	0.069	0.031	-0.01
Quail	Turkey	-2.18	-0.023	0.93	0.015	-0.068	-0.053	0.00
Quail	Ostrich	-2.94	-0.024	-1.61	0.006	0.024	0.031	-0.01
Pheasant	Guinea fowl	-1.31	-0.003	2.46	0.028	-0.053	-0.025	0.12
Pheasant	Turkey	4.28	-0.050	4.92	0.081	-0.190	-0.109	0.13
Pheasant	Ostrich	3.52	-0.051	2.38	0.073	-0.098	-0.026	0.11
Guinea fowl	Turkey	**5.59**	-0.047	2.46	0.053	**-0.137**	-0.084	0.01
Guinea fowl	Ostrich	4.83	-0.048	-0.08	0.044	-0.045	-0.001	-0.00
Turkey	Ostrich	-0.76	-0.001	-2.54	-0.009	0.092	0.083	-0.01

*Bolding indicates significant difference based on Bonferroni threshold of 0.005, for 10 possible species pairwise comparisons within each step category.

Table S3: Post hoc pairwise mean differences between species (column 2 - column 1), in normalised units.

Dependent Variable	Step Type	Species	Obstacle Height	Obstacle Height X Step Type	Species X Step Type
**θ_{TD}**	**663.92**	**6.37**	**9.99**	**105.26**	1.13
**H_{TD}**	**1421.61**	1.32	**8.20**	**223.14**	0.98
**α_{TO}**	**584.10**	**9.00**	1.60	**115.29**	**6.53**
**ΔE_P**	**1402.79**	2.33	**7.13**	**267.98**	**3.01**
**ΔE_K**	**78.83**	**4.57**	**5.37**	**13.99**	2.08
**ΔE_{CoM}**	**217.03**	2.49	**9.39**	**34.40**	**3.58**
**F_{max}**	**114.41**	**16.33**	**9.79**	**19.11**	**2.51**

*Bolding indicates a statistically significant result

Table S4: ANOVA F-statistic results for galliform birds, with obstacle heights from 0.1-0.5L_{leg} (see Methods). Degrees of freedom are as follows: step type = 2; species = 3; obstacle height = 5; obstacle height x step type = 10; species x step type = 6; α_{TO} total = 2360; all other variables total = 2522. Most of the variance in the model is explained by step type and the interaction of obstacle height and step type, reflecting a consistent obstacle negotiation strategy across species. The F-statistics for the effects of step type and obstacle height on leg posture (θ_{TD}, H_{TD}) and potential energy (ΔE_p), which are most indicative of obstacle negotiation strategy, are much larger than the corresponding F-statistics for the effects of species. We did not observe a significant shift in obstacle negotiation strategy with body size between small and large birds (see Supplementary Table S6).

Terrain	θ_{TD} (degrees)	H_{TD}	α_{TO} (degrees)	ΔE_P	ΔE_K	ΔE_{CoM}	F_{max}
**Step -1**							
ObsH=0.1	-1.02	0.027	**1.91**	**0.037**	0.048	**0.085**	0.02
ObsH=0.2	0.16	0.005	**3.98**	**0.085**	0.034	**0.119**	**0.14**
ObsH=0.3	-1.68	0.016	**5.99**	**0.141**	0.066	**0.207**	**0.26**
ObsH=0.4	0.45	0.001	**9.88**	**0.242**	0.041	**0.283**	**0.35**
ObsH=0.5	-1.16	0.011	**11.22**	**0.293**	-0.048	**0.245**	**0.30**
**Step 0**							
ObsH=0.1	**2.78**	**-0.036**	0.33	0.011	-0.042	-0.031	0.02
ObsH=0.2	**7.20**	**-0.101**	-0.07	0.018	-0.039	-0.020	-0.08
ObsH=0.3	**8.16**	**-0.122**	-0.69	0.001	-0.051	-0.050	-0.08
ObsH=0.4	**10.43**	**-0.148**	**-2.75**	-0.014	-0.044	-0.058	**-0.14**
ObsH=0.5	**9.09**	**-0.156**	**-3.99**	**-0.042**	-0.040	**-0.082**	**-0.21**
**Step +1**							
ObsH=0.1	-1.79	**0.051**	-1.60	**-0.043**	0.037	-0.006	0.06
ObsH=0.2	**-2.95**	**0.096**	**-4.24**	**-0.090**	**0.098**	0.008	0.05
ObsH=0.3	**-5.02**	**0.133**	**-4.44**	**-0.113**	**0.090**	-0.023	**0.14**
ObsH=0.4	**-6.54**	**0.181**	**-8.63**	**-0.164**	**0.121**	-0.043	0.13
ObsH=0.5	**-11.08**	**0.222**	**-9.02**	**-0.187**	**0.146**	-0.041	0.10

*Bolding indicates a significant difference based on Bonferroni threshold of 0.0033, for 15 possible obstacle pairwise comparisons within each step category

Table S5: Post hoc pairwise mean differences (Obs- Level) in normalised units, for obstacle heights by step type across galliform birds.

Species 1	Species 2	θ_{TD} (degrees)	H_{TD}	α_{TO} (degrees)	ΔE_P	ΔE_K	ΔE_{CoM}	F_{max}
**Step -1**								
Quail	Pheasant	--	--	-1.91	0.015	--	0.060	**-0.28**
Quail	Guinea fowl	--	--	-0.63	-0.000	--	0.021	-0.07
Quail	Turkey	--	--	-0.31	0.011	--	0.013	-0.01
Pheasant	Guinea fowl	--	--	1.28	-0.015	--	-0.039	**0.21**
Pheasant	Turkey	--	--	1.60	-0.004	--	-0.047	**0.27**
Guinea fowl	Turkey	--	--	0.32	0.011	--	-0.008	0.06
**Step 0**								
Quail	Pheasant	--	--	0.14	-0.012	--	-0.037	**-0.26**
Quail	Guinea fowl	--	--	1.33	-0.010	--	-0.013	-0.05
Quail	Turkey	--	--	0.64	-0.018	--	**-0.062**	0.06
Pheasant	Guinea fowl	--	--	1.19	0.003	--	0.024	**0.21**
Pheasant	Turkey	--	--	0.50	-0.005	--	-0.025	**0.31**
Guinea fowl	Turkey	--	--	-0.69	-0.008	--	**-0.049**	**0.11**
**Step +1**								
Quail	Pheasant	--	--	-1.97	0.000	--	0.005	**-0.32**
Quail	Guinea fowl	--	--	**-1.73**	-0.016	--	-0.001	-0.03
Quail	Turkey	--	--	**-1.89**	-0.013	--	-0.019	-0.04
Pheasant	Guinea fowl	--	--	0.24	-0.016	--	-0.006	**0.29**
Pheasant	Turkey	--	--	0.08	-0.013	--	-0.024	**0.28**
Guinea fowl	Turkey	--	--	0.16	0.003	--	-0.019	-0.01

*Bolding indicates significant difference based on Bonferroni threshold of 0.0083, for 6 possible species pairwise comparisons within each step category.

Table S6: Post hoc pairwise mean differences between galliform species (column 2-column 1) from ANOVA (Table S4). Notably, pairwise differences in leg posture (θ_{TD}, H_{TD}) and change in potential energy (ΔE_p), which are most indicative of obstacle negotiation strategy, do not significantly differ between species.

	Species				
	Quail	Pheasant	Guinea fowl	Turkey	Ostrich
**Fitted Parameters**					
Spring stiffness ($k_{norm}=k*L_{leg}/(m*g)$)	8.0	11	15	10	12
Damping coefficient $\left(c_{norm} = \frac{c}{m}\sqrt{\frac{L_{leg}}{g}}\right)$	0.10	0.20	0.40	0.20	0.40
**Computed Property**					
Damping ratio $\left(\zeta = \frac{c}{2\sqrt{km}}\right)$	0.018	0.020	0.052	0.032	0.058
**Optimal trajectory performance**					
Mean-squared error	0.0102	0.0266	0.0032	0.0081	0.0063
Net unsigned work (Joules / ($m*g*L_{leg}$))	0.3149	0.1708	0.0992	0.0761	0.0081
**Normalising Parameters**					
m (kg)	0.200	1.02	1.48	2.96	116
L_{leg} (m)	0.117	0.201	0.228	0.287	0.974
g (m/s^2)			9.81		

Table S7: Normalised results of trajectory optimisation applied to the actuated model (Fig. 6A), resulting in the reported fits to bird GRF (Fig. 6B) and leg length trajectories (Fig. 6C). Bird size spanned over a 500-fold mass range, but the damping ratio remained with a factor of 3.27 across species. Average masses reported in this table differ somewhat from those reported in main text because here the mass averaging was weighted by number of level step samples, not by individual birds. Given the non-unique nature of the parameter fits (Fig. S3), we do not make scientific claims about the functional significance of any one particular set of parameters. Nonetheless, a relatively narrow range of damping ratios results in fits consistent with bird running data.
